# Health Tract, No. 294

**Published:** 1867-08

**Authors:** 


HEALTH TRACT, No. 294.

### MARRIAGE AND LONGEVITY.

Bachelors die earlier than other men, see Tract 290, on " Woman." This is confirmed by Dr.Stark of the Registers office in Scotland, who finds that the average age of married men over twenty years is over fifty-nine years, while the unmarried average only forty years ; that is, marriage adds nearly one-third to the length of life, as a general rule, because

1st. Bachelors are always in a state of unrest, they feel unsettled.

2nd. If indoors after supper there is a sense of solitariness, inducing a sadness, not actual melancholy, with all their depressing influences ; and many, many hours in the course of the year are spent in gloomy inactivity, which is adverse to a good digestion and a vigorous and healthful circulation.

3d. His own chamber or house being so uninviting, the bachelor is inclined to seek diversion outside, in suppers with friends, in clubs which are introductories to intemperance and licentiousness, or to those more unblushing associations which under the cover of darkness, lead to speedy ruin of health and morals; and when these are gone the way downward to an untimely grave is rapid and certain.

On the other hand, marriage lengthens a man's life.

1st. By its making home inviting.

2nd. By the softening influences which it has upon the character and the affections.

3d. By the cultivation of all the better feelings of our nature, and in that proportion saving from vice and crime.

4th. There can be no healthful development of the physical functions of our nature without marriage ; it is necessary to the perfect man, for Divinity has announced that it was "not good for a man to be alone."

5th. Marriage gives a laudable and happyfying object in life, the provision for wife and children, their present comfort and future welfare, the enjoyment in witnessing their happiness and the daily and hourly participations in affectionate interchange of thought and sentiment and sympathy, these are the considerations which antagonize sorrow and lighten the burdens of life, thus strewing flowers and casting sunshine all along its pathway.

